# Clinical Insights into Hearing Loss

**DOI:** 10.3390/audiolres15040107

**Published:** 2025-08-13

**Authors:** George Psillas, Petros D. Karkos

**Affiliations:** 1st Academic ENT Department, Aristotle University of Thessaloniki, AHEPA Hospital, 1, Stilponos Kyriakidi St., 546 36 Thessaloniki, Greece

We are pleased to present a Special Issue addressing new insights into the causes, symptoms, diagnosis, and treatment of hearing loss.

This Special Issue comprises twelve publications that cover topics relating to hearing aids, auditory neuropathy, implantable auditory devices such as cochlear implants, and middle ear and bone conduction implants. In addition, congenital cytomegalovirus-related hearing loss and cisplatin-induced ototoxicity are discussed in the form of a review. Lastly, the effects of different hyperbaric oxygen therapy profiles on hearing improvement are evaluated in patients suffering from sudden sensorineural hearing loss.

Despite technological advancements, hearing aid users still face difficulty in understanding speech in noise. Research primarily focuses on assessing parameters that contribute to the evaluation of patients prior to hearing aid use. The Acceptable Noise Level is a key factor influencing hearing aid fittings, determining how much background noise can be tolerated before it interferes with speech intelligibility. In a multicenter study conducted by Mark Laureyns and colleagues [1], the authors evaluated the influence of the masker multi-talker babble on the Acceptable Noise Level; it was concluded that high-frequency environmental noise is the most important condition affecting Acceptable Noise Level results, regardless of the grade of hearing impairment.

It is recognized that better unaided speech-in-noise performance is associated with greater perceived benefit from hearing aids. The association between unaided speech-in-noise performance and hearing aid use, mediated through perceived benefit, had not been previously studied. Anthony Marcotti and colleagues [2] found no direct association between unaided speech-in-noise performance and hearing aid use; however, unaided speech-in-noise performance, particularly in the left ear, should be considered during the hearing aid fitting process.

Although the correct fitting of hearing aids may be achieved, this is not sufficient; consistent and effective use is essential for success in elderly adults. Abdolhakim Jorbonyan and colleagues [3] demonstrated in Persian adult participants that the use of questionnaires, such as MARS-HA, may provide a better understanding of the level of self-efficacy in using hearing aids; higher self-efficacy in the technical-auditory and psychosocial field was associated with greater hearing aid acceptance and successful use.

In general, hearing-impaired people prefer hearing aids that match their skin color to be less noticeable in their environment. However, over-the-counter hearing aids are only widely available in relatively lighter beige colors, with limited availability for BIPOC (Black, Indigenous, and People of Color) individuals with darker skin colors; this fact may contribute to a relatively inequitable over-the-counter hearing aid device offering to individuals who have mild-to-moderate hearing loss and darker skin colors [4].

Auditory processing disorder in children is a condition in which the brain has difficulty processing and interpreting sounds, particularly speech. Questionnaires and behavioral checklists have been reported in the international literature to pinpoint those children who may be susceptible to auditory processing disorder. The Greek version of these questionnaires has been applied in Greek Cypriot-speaking children [5], and they have aided in identifying children at risk of auditory processing disorder, providing a valuable tool for family counselling.

Congenital cytomegalovirus-related hearing loss is the most prevalent non-genetic cause of pediatric sensorineural hearing loss [6]. Nicoleta Gana and colleagues [7], in a detailed review, described the diagnostic approaches and management strategies employed for congenital cytomegalovirus-related hearing loss; they underlined the need for prompt detection of hearing loss and interdisciplinary care for treatment.

Up to 93% of patients undergoing cisplatin chemotherapy may develop hearing loss, which may result in a decreased quality of life in cancer survivors [8]. In their study, Alexandru Orasan and colleagues [9] reported on otoprotective substances, including antioxidants, anti-inflammatory agents, apoptosis inhibitors, intratympanic injection of N-acetylcysteine, and nanoparticle-based systems; sound conditioning and epigenetic drugs may be used to prevent hearing loss and modulate gene expression.

The treatment of hearing loss is one of the main challenges addressed in this Special Issue, and five papers are devoted specifically to this topic. First, Pawel Rozbicki and colleagues [10] underlined the beneficial effect of the early initation (less than 10 days) of hyperbaric oxygen in up to 15 cycles for the treatment of sudden sensorineural hearing loss. Joan Lorente-Piera and colleagues [11] demonstrated the effectiveness of Vibrant Soundbridge for the treatment of conductive, mixed, or sensorineural hearing loss, especially at higher frequencies; notable hearing amplification was achieved with the placement of the floating mass transducer in the coupling of round and oval windows, ensuring the more efficient transmission of vibrations to the cochlear fluids. Oana Astefanei and colleagues [12] in their comprehensive article concluded that both cochlear implants and bone conduction implants may have a beneficial effect in children and adults with single-sided deafness and asymmetric hearing loss. Mastoid cavity obliteration with the use of a temporoparietal fascial flap injected using injectable platelet-rich fibrin in patients who underwent subtotal petrosectomy for cochlear implantation was first described by Aleksander Zwierz and colleagues [13]. Using a mouse model, David Bächinger and colleagues [14] reported electrophysiological findings that may help to enhance hearing preservation during cochlear implantation; they provided evidence indicating that the more basally a cochlear trauma is located, the larger the cochlear microphonic amplitude drops.

There are a number of gaps in our knowledge in the field of audiology and otology, requiring continued research into early diagnosis, intervention, and individualized strategies. We hope that the articles published in this Special Issue advance our understanding of hearing loss and provide an update on new aspects of clinical practices, novel therapeutic approaches, and surgical techniques.
